# LncHOXA10 drives liver TICs self-renewal and tumorigenesis via HOXA10 transcription activation

**DOI:** 10.1186/s12943-018-0921-y

**Published:** 2018-12-13

**Authors:** Ming Shao, Qiankun Yang, Weitao Zhu, Huifang Jin, Jing Wang, Jie Song, Yongkui Kong, Xianping Lv

**Affiliations:** 1 Department of Blood Transfusion, The First Affiliated Hospital of Zhengzhou University, Zhengzhou, 450052 Henan Province China; 2 Department of Clinical Laboratory, The First Affiliated Hospital of Zhengzhou University, Zhengzhou, 450052 Henan Province China

**Keywords:** Liver cancer, Tumor initiating cells, Long noncoding RNA, HOX transcription factor

## Abstract

**Background:**

Liver cancer is one of the most deadly cancers in the world. There are various cells in liver tumor bulk, including liver tumor initiating cells (TICs), which account for liver tumorigenesis, drug resistance, relapse and metastasis. The homeobox (HOX) transcription factors play critical roles in many physiological and pathological processes, while, their roles in liver TICs and liver tumorigenesis remain unknown.

**Methods:**

An unbiased screening was performed using online-available datasets. Liver TICs were sorted by FACS using surface markers CD133, CD13 and EPCAM, or enriched by oncosphere formation assay. TIC self-renewal was examined by oncosphere formation and tumor initiation assay. Loss of function and gain of function assays were performed to examine the role of lncRNA. RNA pulldown, RNA immunoprecipitation, ChIP, Western blot and double FISH were used to explore the molecular mechanism of lncRNA.

**Results:**

Here, we examined the expression pattern of HOX transcription factors, and found HOXA10 was overexpressed in liver cancer samples. Moreover, a divergent lncRNA of HOXA10 (termed lncHOXA10 hereafter) was also highly expressed in liver cancer and liver TICs. LncHOXA10 drove liver TIC self-renewal and liver tumorigenesis through HOXA10-dependent manner. LncHOXA10 interacted with SNF2L and recruited NURF chromatin remodeling complex to *HOXA10* promoter, and thus initiated the transcription of HOXA10. Through HOXA10 transcriptional regulation, lncHOXA10 activated HOXA10 in liver TICs. LncHOXA10-HOXA10 signaling can be targeted to eliminate liver TICs. Altogether, lncHOXA10 drove HOXA10 expression and thus promoted liver TIC self-renewal.

**Conclusion:**

HOXA10 was the most highly expressed HOX transcription factor in liver cancer and liver TICs. LncHOXA10 drove the transcriptional activation of HOXA10. This work revealed the important role of HOX transcription factor in liver TIC self-renewal and added a new layer for liver TIC regulation.

**Electronic supplementary material:**

The online version of this article (10.1186/s12943-018-0921-y) contains supplementary material, which is available to authorized users.

## Background

Liver cancer is one of the most serious cancers all over the world, leading to hundreds of thousands of deaths. Liver cancer contains two common cancer types, hepatocellular carcinoma (HCC, about 90%) and cholangiocarcinoma (CC, about 10%). There are various kinds of cells in liver tumor bulk and only a small subset of cells can generate new tumors efficiently, which are termed liver tumor initiating cells (TICs). Liver TICs account for liver tumorigenesis, metastasis, drug-resistance and relapse [1]. With self-renewal and differentiation capacities, liver TICs can relapse and generate new tumors when drugs are stopped [2, 3]. Several functional assays for liver TICs have been established, including sphere formation assay for liver TIC self-renewal, side-population assay for drug-resistance, gradient xenograft assay for tumorigenesis and propagation, transwell assay for metastasis and invasion [4–6]. Among these assays, gradient xenograft and sphere formation assays are widely-used for liver TIC determination. For liver TIC detection and enrichment, several surface markers of liver TICs have been identified, including CD133, CD13, EPCAM, CD24, CD90 and so on [7–9]. Despite of the importance of liver TICs in liver tumorigenesis and treatment, their biological characteristics remain elusive.

The self-renewal of liver TICs is finely regulated by many modulators. Wnt/β-catenin signaling, Notch signaling, Hedgehog signaling, PKC signaling and Yap1 signaling are the most important regulators in liver TIC regulation [10–13]. Some transcription factors (TF) also participate in the self-renewal regulation of liver TICs, including Zic2, Oct4, Sox4, Notch2 [12, 14, 15]. Homeobox (HOX) transcription factors participate in many physiological and pathological processes, including vertebrate development [16], neural crest and branchial arch patterning [17], angiogenesis [18] and tumorigenesis [19]. However, the roles of HOX TFs in liver TIC self-renewal are unclear. Through unbiased screening, here we examined the expression profile of HOX TFs in liver cancer, and found HOXA10 (homeobox A10) was the most highly expressed HOX transcription factor in liver tumor.

HOXA10 is a member of HOX transcription factor family. HOX TFs are grouped in four clusters (cluster A, B, C and D). HOXA10, located in A cluster on chromosome 7, plays critical roles in gene expression, morphogenesis, and differentiation. HOXA10 also participates in fertility, embryo viability and hematopoietic lineage commitment. Of interest, HOXA10 crosses with other signaling pathways, including Wnt/β-catenin signaling [20], PI3K signaling [21], TGFβ signaling [22] and so on. As for tumorigenesis, HOXA10 participates in ovarian clear cell adenocarcinoma [23], oral squamous cell carcinoma [24], prostate carcinoma [25], gastric cancer [26], endometrial adenocarcinoma [27] and so on. While, its role in liver tumorigenesis and liver TICs is unclear. Here we found HOXA10 was up-regulated in liver tumorigenesis and TIC self-renewal. HOXA10 knockout cells showed impaired self-renewal capacity.

Long noncoding RNAs (lncRNAs) are defined as RNA transcripts longer than 200 nucleotides without protein coding potential [28]. Recent studies reveal lncRNAs as important modulators in many biological processes, including tumorigenesis [29]. LncRNAs participate in tumor formation, energy metabolism, colony formation, metastasis and so on [30–33]. LncRNAs exert their roles *in trans* or *in cis*. Many lncRNAs interact with epigenetic complexes (SWI/SNF, NURF, NURD, PRC1/2 and so on) and recruit them to the promoter of target genes [29]. LncRNAs also combine with RNA-binding proteins and change the stability or activity of their partners [31, 34]. Here, we found lncHOXA10 and HOXA10 are highly expressed in liver cancer and liver TICs. LncHOXA10 and HOXA10 are involved in the self-renewal regulation of liver TICs. LncHOXA10 interacts and recruits NURF chromatin remodeling complex to *HOXA10* promoter to drive its transcription initiation. *LncHOXA10*-HOXA10 pathway can be used for liver TIC targeting.

## Methods

### Primary samples

HCC primary samples were obtained from the first affiliated hospital of Zhengzhou University with informed consent (number: 2016–01-013C). All human and mouse experiments were approved by the Institutional Committee of Zhengzhou University. HCC samples were numbered by the obtaining time, and some samples (#1, #2, #4, #5, #7, #9) with sphere formation capacity were selected for experiments. The details for these sample were: #1, advanced hepatocellular carcinoma, 72 years old, male, the size of tumor bulk used in this work, 7.1 × 6.2 × 5.4 mm, non-metastasis. #2, advanced hepatocellular carcinoma, 68 years old, male, tumor bulk size, 7.3 × 6.1 × 4.9 mm, non-metastasis. #4, advanced hepatocellular carcinoma, 63 years old, female, tumor bulk size, 6.8 × 5.1 × 4.3 mm, non-metastasis. #5, early hepatocellular carcinoma, 58 years old, male, tumor bulk size, 4.2 × 3.8 × 2.9 mm, non-metastasis. #7, advanced hepatocellular carcinoma, 75 years old, female, tumor bulk size, 10.7 × 9.1 × 8.2 mm, metastasis. #9, advanced hepatocellular carcinoma, 68 years old, female, tumor bulk size, 9.8 × 7.2 × 6.1 mm, metastasis.

### Antibodies and reagents

Anti-β-actin antibody (cat. no. A1978) and DAPI (cat. no. 28718–90-3) were purchased from Sigma-Aldrich. Anti-HOXA10 (cat. no. 26497–1-AP) antibody was from Proteintech. Anti-SNF2L (cat. no. HPA003335) antibody was obtained from Atlas Antibodies. Phycoerythrin (PE)-conjugated CD133 (cat. no. 130098826) was obtained from MiltenyiBiotec. Fluorescence-conjugated secondary antibodies were purchased from Molecular Probes. The LightShift Chemiluminescent RNA EMSA (cat. no. 20158) and Chemiluminescent Nucleic Acid Detection kits (cat. no. 89880) were from Thermo Scientific. T7 RNA polymerase (cat. no. 10881767001), Biotin RNA Labeling Mix (cat. no. 11685597910) were obtained from Roche.

### Oncosphere formation

DMEM/F12 medium, supplemented with N2, B27, 20 ng/ml EGF and 20 ng/ml bFGF was used for oncosphere formation. 5000 HCC cells were incubated in oncosphere medium and seeded into Ultra Low Attachment 6-well plates for 2 weeks. Spheres and non-spheres can be separated according to size difference. The materials for sphere formation were purchased from: bFGF (Millipore, cat. no. GF446-50UG), EGF (Life Technologies, cat. no. E5036-200UG), N2 supplement (Life Technologies, cat. no. 17502–048), B27 (Life Technologies, cat. no. 17504–044) and Ultra low attachment plates (Corning, cat. no. 3471).

### Tumor propagation and initiation assay

For tumor propagation, 1 × 10^6^ lncHOXA10 silenced and control cells were injected into 6-week-old BALB/c nude mice for one-month tumor propagation. For tumor-initiation detection, gradient (10, 1 × 10^2^, 1 × 10^3^, 1 × 10^4^, and 1 × 10^5^) lncHOXA10 silenced cells and control cells were injected into 6-week-old BALB/c nude mice for 3 month tumor initiation. Six mice were used for each sample.

### RNA pulldown

Biotin-labeled lncHOXA10 were obtained in vitro by biotin RNA labeling mix (Roche). Then the labeled transcripts were incubated with sphere lysate. Streptavidin beads were added and the enriched components were analyzed by SDS PAGE, followed by Western blot or mass spectra.

### CRISPR/Cas9 knockout

*HOXA10* knockout cells were established using CRISPR/Cas9 lentivirus. Briefly, sgRNA targeting HOXA10 was designed according to CRISPR design tool (http://crispr.mit.edu/) and cloned into LentiCRISPRv2 vector. Two sgRNAs were generated and used for lentivirus generation. LentiCRISPRv2, pVSVg and psPAX2 were transfected into 293 T cells for lentivirus package. 48 h later, lentivirus was collected to infect HCC primary cells, followed by puromycin selection. Five days later, infected cells were collected for Western blot to detect the efficiency of *HOXA10* knockout. Empty lentiCRISPRv2 also used for lentivirus package, and infected cells were used as WT control. The sequences of two sgRNAs used for *HOXA10* knockout were 5′- CCAAAAAAGAGTTCGCGGCG-3′ and 5’-CGGTTACTACGCCCACGGCG-3′.

### RNA antisense purification

RNA antisense purification (RAP) was performed to identify the interaction protein of endogenous lncHOXA10. For RAP, overlying DNA probes were designed and incubated with sphere lysate individually, and treated with RNase H. If a probe targets endogenous lncHOXA10 efficiently, DNA/RNA complex will be formed and lncHOXA10 will be digested by RNase H. Otherwise, a probe can’t target endogenous lncHOXA10 induces intact lncHOXA10. The binding capacity of lncHOXA10 probes can be distinguished by Northern blot. Non-targeting probes were grouped into Probeset #1 and binding probes were grouped into Probeset #2. Biotin labeled Probeset #1 and #2 were incubated with sphere lysate, and the binding partners were enriched by streptavidin conjugated Beads. The eluate samples were analyzed by silver staining or Western blot.

### Immunohistochemistry

Formalin-fixed liver cancer sections were treated with xylene and graded alcohols, followed by 15 min’s incubation in 3% Hydrogen Peroxide (H_2_O_2_). Then the sections were boiled 15 min in Tris/EDTA buffer for antigen retrieval. Then the samples were incubated in primary and secondary antibodies, followed by treatment with HRP substrate. Nikon-EclipseTi microscopy was used for observation.

### Chromosome immunoprecipitation (ChIP)

ChIP assays were performed according to the manual of Upstate Biotechnology. Briefly, lncHOXA10 silenced spheres were treated with 1% formaldehyde at 37 °C for crosslinking and crushed by SDS lysis buffer, followed by ultrasonic treatment to shear DNA. SNF2L, HeK4me3 and RNA pol II antibodies were added into mixture for DNA segment enrichment. Finally the enrichment of *HOXA10* promoter was detected by realtime PCR with ABI7300.

### Statistical analysis

One-tailed Student’s t tests were used for statistical analysis. *P* < 0.05 was considered to be statistically significant.

## Results

### HOXA10 was highly in liver cancer and liver TICs

**Fig. 1:**
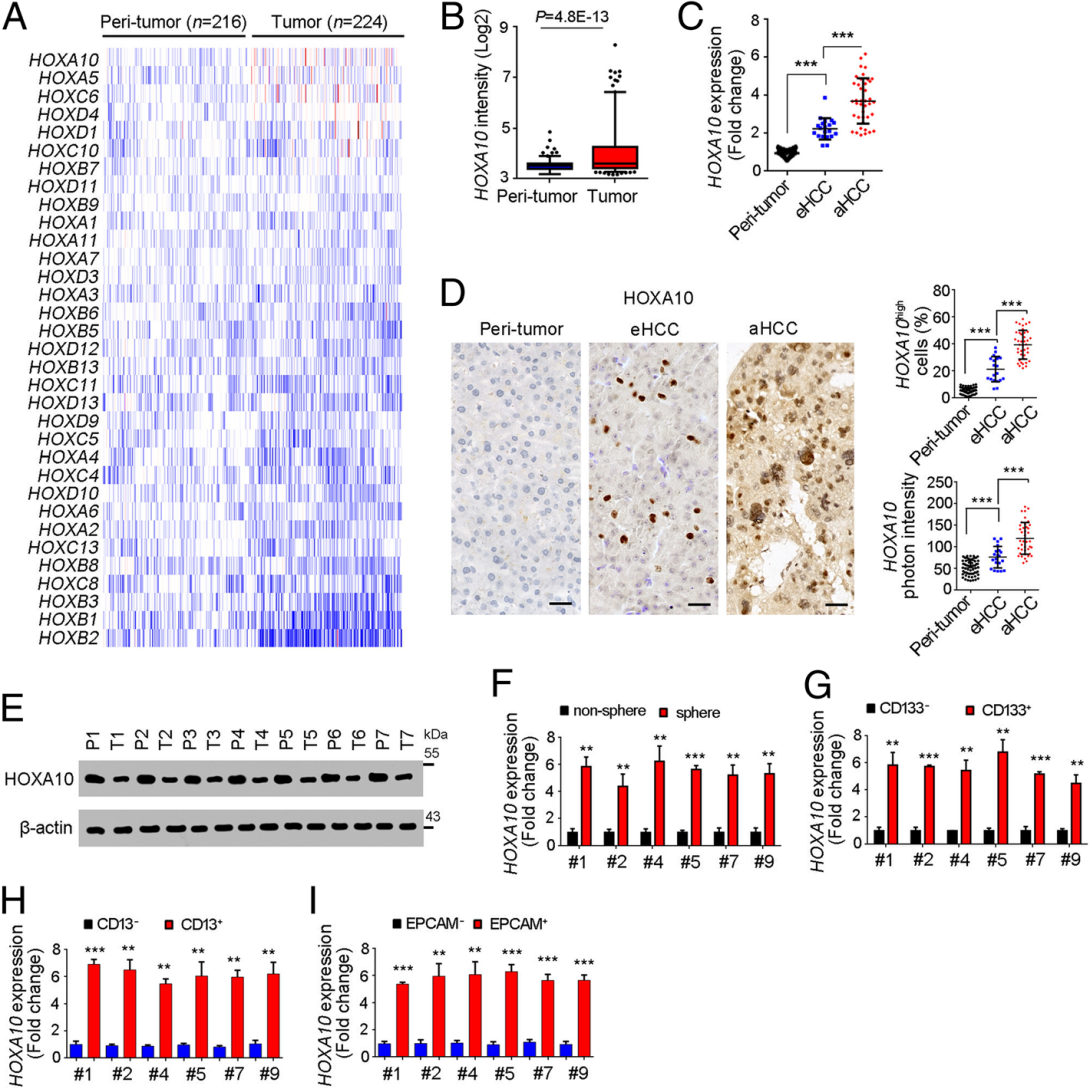
High expression of HOXA10 in liver cancer and liver TICs. **a**, **b** Expression profiles of HOX transcription factors were shown as heatmap. GSE14520 containing 214 peri-tumor samples and 224 tumor samples was analyzed. **b** HOXA10 expression profiles in peri-tumor and tumor samples were analyzed. **c** HOXA10 expression was analyzed in 60 peri-tumor samples, 20 early HCC samples (eHCC) and 40 advanced HCC samples (aHCC). All expression results were normalized to the average of peri-tumor expression and shown as scatter diagram. **d** Immunohistochemistry of HOXA10 in peri-tumor, eHCC and aHCC. Typical images were shown in left panels and calculated ratios were shown in right panels. **e** Western blot confirmed the high expression of HOXA10 in liver cancer. **f** HOXA10 expression in oncospheres and non-spheres was examine with realtime PCR. **g**-**i** CD133^+^ TICs (**g**), CD13^+^ TICs (**h**), EPCAM^+^ TICs (I) and corresponding non-TICs were collected for mRNA extraction, and HOXA10 expression levels were examined by realtime PCR. All expression results were normalized to non-TIC expression levels. For B, data were shown as box and whisker plot (Whiskers are 5th and 95th percentiles). For C, D, F, G, H, I, data were shown as means ± s.d. *P < 0.05; **P < 0.01; ***P < 0.001 by one-tailed Student’s t test. Data are representative of four independent experiments

Liver cancer is one of the most serious cancers all over the world. HOX TFs participate in many biological processes. However, the roles of HOX TFs in liver tumorigenesis and liver TICs are elusive. Taking advantage of online-available dataset (GSE14520) [35, 36], we performed a unbiased screening for the expression levels of HOX TFs in liver cancers. Of the denoted HOX TFs, HOXA10 was the highest expressed HOX transcription factor in liver cancer (Fig. 1a, b).

We then collected primary cells and examined the expression levels of HOXA10 with realtime PCR (Fig. 1c), immunohistochemistry (Fig. 1d) and Western blot (Fig. 1e), confirming the high expression of HOXA10 in liver cancer. Moreover, HOXA10 expression levels were also related to clinical severity of liver cancer (Fig. 1c, d).

To examine the expression of HOXA10 in liver TICs, we performed sphere formation assay, and found HOXA10 was highly expressed in oncospheres (Fig. 1f). We also enriched liver TICs by FACS using surface marker CD133, CD13 and EPCAM, and confirmed high expression of HOXA10 in liver TICs (Fig. 1g-i). Altogether, HOXA10 was up-regulated in liver cancer and liver TICs.

### High expression of lncHOXA10 in liver cancer and liver TICs

**Fig. 2:**
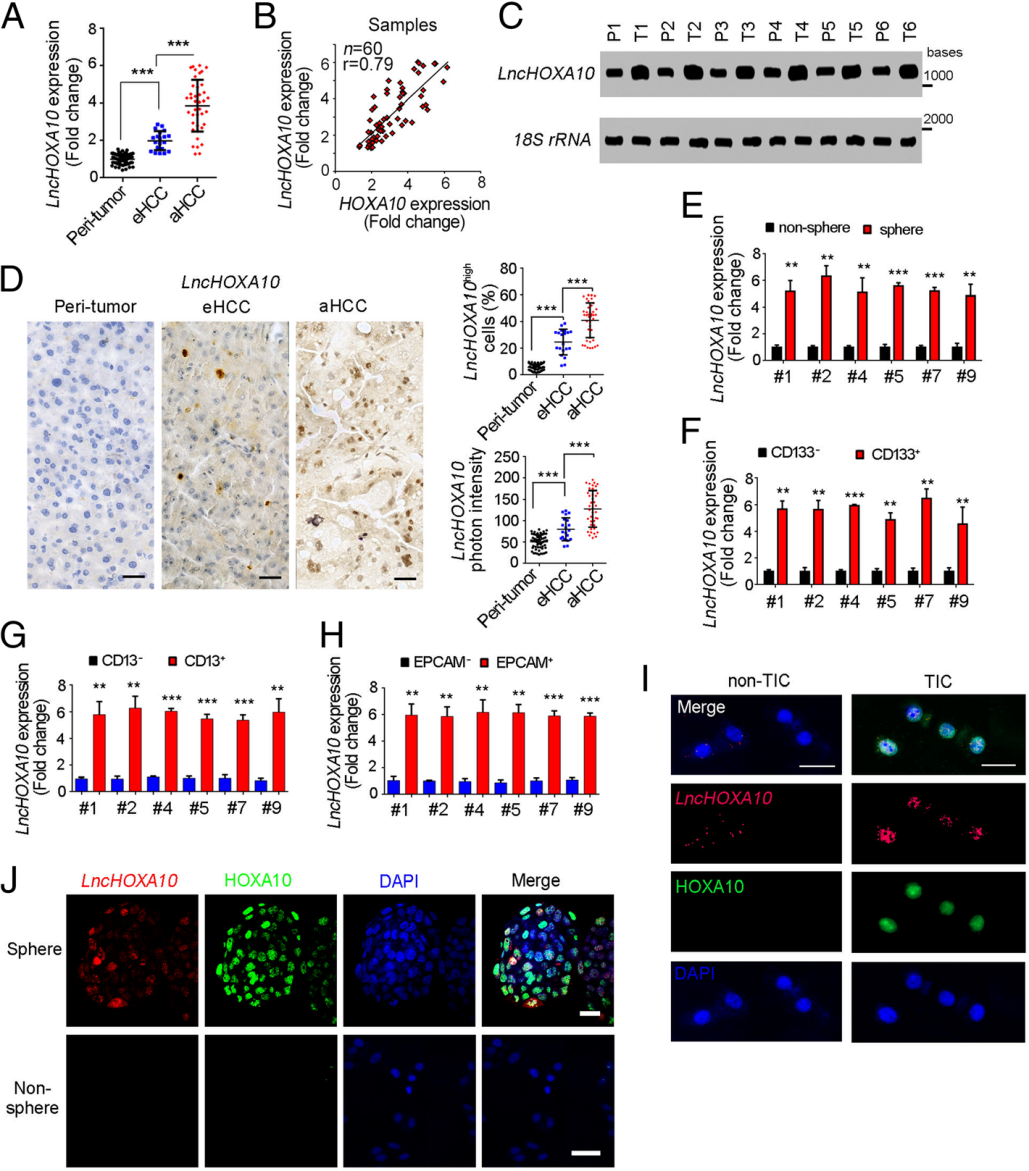
LncHOXA10 was highly expressed in liver cancer and liver TICs. **a** LncHOXA10 expression levels in 60 peri-tumor, 20 early HCC and 40 advanced HCC samples were analyzed through realtime PCR. **b** Positive correlation between HOXA10 and lncHOXA10 was shown. **c** LncHOXA10 expression levels were detected through Northern blot. 18S rRNA was a loading control. **d** In situ hybridization (ISH) of lncHOXA10 in peri-tumor, eHCC and aHCC samples. 60 peri-tumor, 20 early HCC and 40 advanced HCC samples were used. Typical images were shown in left panels and indicated ratios were shown in right panels. **e** Spheres and non-spheres were collected, and lncHOXA10 expression levels were detected through realtime PCR. All expression levels were normalized to the average expression levels of non-sphere samples. **f**-**h** CD133^+^ (**f**), CD13^+^ TICs (**g**), EPCAM^+^ TICs (**h**) and non-TICs were enriched by FACS, followed by realtime PCR examination for lncHOXA10 expression. **i**, **j** Fluorescence in situ hybridization (FISH) of lncHOXA10. CD133^+^ liver TICs (**i**) and oncospheres (**j**) were used. Scale bars, D, 50 μm; I, J, 20 μm. Data were shown as means ± s.d. *P < 0.05; **P < 0.01; ***P < 0.001 by one-tailed Student’s t test. Data are representative of three independent experiments

We then investigated the regulation mechanism of HOXA10 expression. Firstly, we focused on *HOXA10* locus, and found lncRNA ENST00000519935.1 (hereafter termed lncHOXA10) near *HOXA10* locus. Through realtime PCR, we found lncHOXA10 was highly expressed in liver cancer (Fig. 2a). Of interest, lncHOXA10 expression was positively related to HOXA10 expression (Fig. 2b). We then examined lncHOXA10 expression levels through Northern blot (Fig. 2c) and in situ hybridization (Fig. 2d), confirming an increased expression of lncHOXA10 in liver tumor.

We also examined the expression levels of lncHOXA10 in liver TICs. We examined an increased lncHOXA10 expression in oncospheres (Fig. 2e) and liver TICs (Fig. 2f-h). We also stained lncHOXA10 and HOXA10 in liver TICs (Fig. 2i) and spheres (Fig. 2j), and validated high expression of lncHOXA10 and HOXA10 in liver TICs and oncospheres. Taken together, lncHOXA10 was highly expressed in liver cancer and liver TICs.

### LncHOXA10 drove the self-renewal of liver TICs

**Fig. 3:**
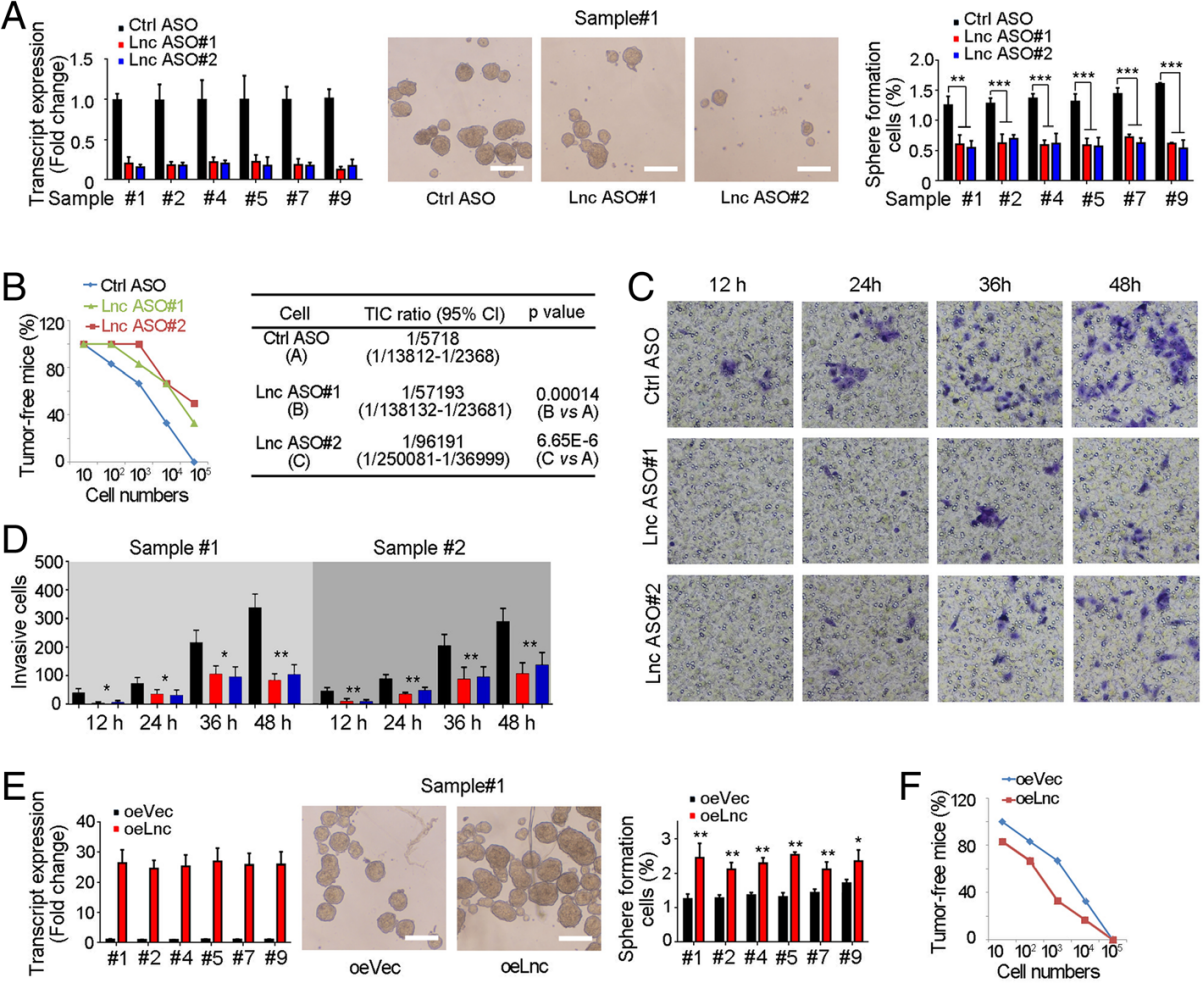
LncHOXA10 played an essential role in liver TIC self-renewal. **a** LncHOXA10 depleted cells were generated with antisense oligo (ASO) and sphere formation assays were performed. LncHOXA10 depletion was confirmed by realtime PCR (left panels). Sphere photos were shown in middle panels and sphere-initiating ratios were shown in right panels. **b** 10, 1 × 10^2^, 1 × 10^3^, 1 × 10^4^ and 1 × 10^5^ lncHOXA10 silenced cells and control cells were injected into BALB/c nude mice for 3-months’ tumor initiation. The ratios of tumor-free mice were shown in left panel and TIC ratios were calculated by extreme limiting dilution analysis (right panels). CI, confidence interval. **c**, **d** LncHOXA10 silenced cells were used for transwell invasion assay. At the indicated time points, invasive cells were visualized by crystal violet staining. Typical images (**c**) and invasive cell numbers (**d**) were shown. **e** LncHOXA10 overexpressing cells were established for sphere formation assays. Enhanced sphere formation capacity was observed in lncHOXA10 overexpressing cells. **f** Gradient numbers of lncHOXA10 overexpressing cells were used for 3-months’ tumor initiation and the ratios of tumor-free mice were shown. *P < 0.05; **P < 0.01; ***P < 0.001 by one-tailed Student’s t test. Data are representative of four independent experiments

We then explored the role of lncHOXA10 in liver TIC self-renewal. LncHOXA10 antisense oligo (ASO) treatment largely impaired oncosphere formation, indicating the critical role of lncHOXA10 in liver TIC self-renewal (Fig. 3a). We also injected gradient numbers of lncHOXA10 depleted cells into BALB/c nude mice, and observed impaired tumor initiation and decreased liver TIC ratios upon lncHOXA10 depletion (Fig. 3b). Using transwell assay, we also confirmed the essential role of lncHOXA10 in the invasion of liver TICs (Fig. 3c, d).

We then generated lncHOXA10 overexpressing cells and found enhanced sphere formation, confirming the critical role of lncHOXA10 in liver TIC self-renewal (Fig. 3e). Then we used lncHOXA10 overexpressing cells for tumor initiation, and confirmed the critical role of lncHOXA10 in liver tumor initiation (Fig. 3f). Taken together, lncHOXA10 drove the self-renewal of liver TICs.

### LncHOXA10 promoted HOXA10 expression

**Fig. 4:**
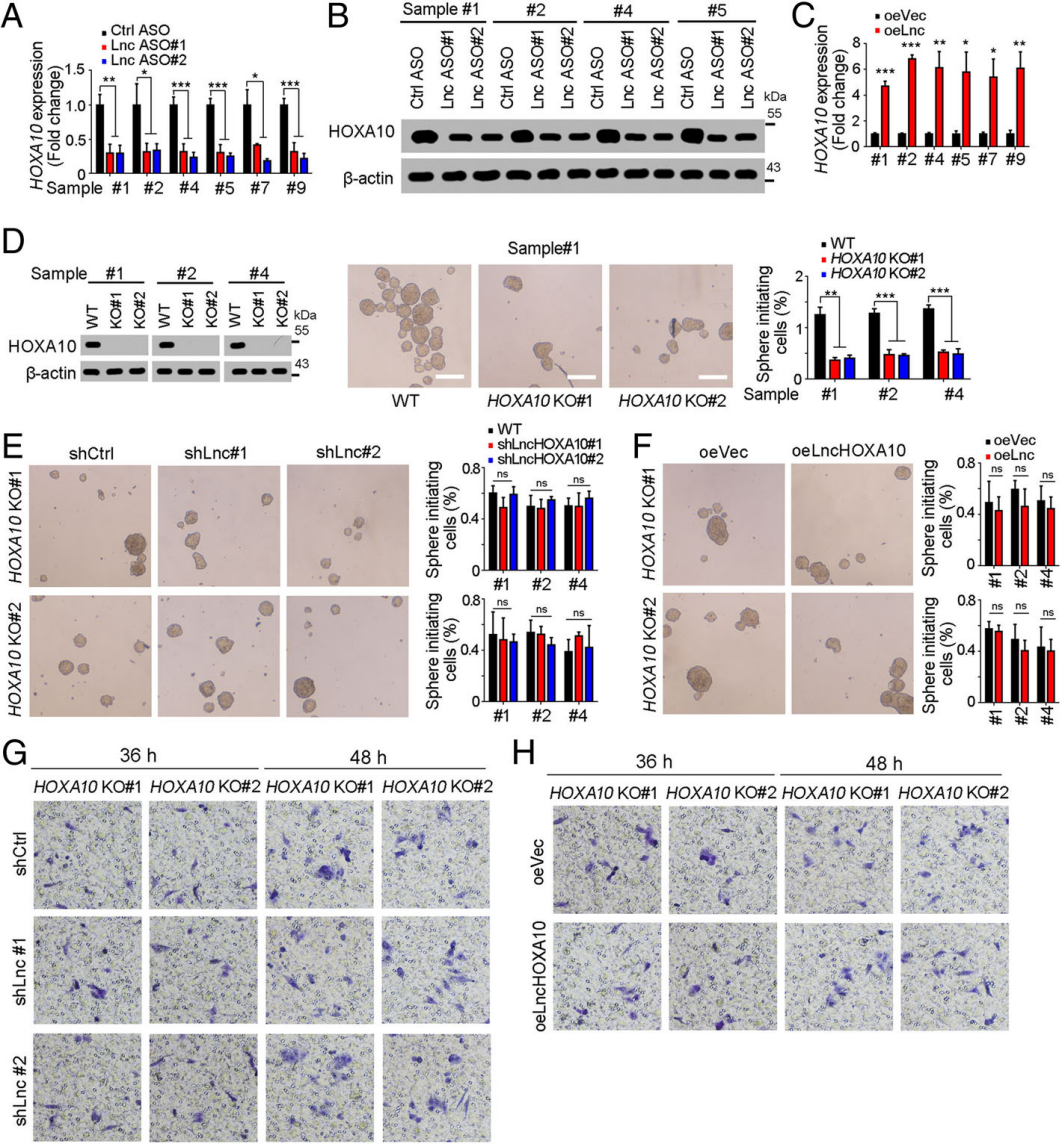
LncHOXA10 promoted HOXA10 expression. **a**, **b** HOXA10 expression levels in lncHOXA10 depleted cells were examined through realtime PCR (**a**) and Western blot (**b**). β-actin served as a loading control. **c** LncHOXA10 overexpressing cells were used for HOXA10 examination. **d** *HOXA10* knockout cells were established through CRISPR/Cas9 approach, followed by sphere formation. Typical sphere images and sphere-initiating ratios were shown in middle and right panels, respectively. **e**, **f** LncHOXA10 was silenced (**e**) or overexpressed (**f**) in *HOXA10* knockout cells, followed by sphere formation assay. LncHOXA10 had an impaired role in liver TIC self-renewal upon *HOXA10* knockout. **g**, **h** *HOXA10* knockout cells were used for lncHOXA10 knockdown (**g**) and overexpression (**h**), followed by transwell invasion assay. Invasive cells were detected at the indicated time points and typical images were shown. *P < 0.05; **P < 0.01; ***P < 0.001; ns, not significant, by one-tailed Student’s t test. Data are representative of three independent experiments

It’s a common mechanism that lncRNAs participate in the transcription process of nearby genes. Here we found the expression levels of lncHOXA10 and HOXA10 were positively correlated, thus we examined whether lncHOXA10 drove HOXA10 expression. Decreased HOXA10 expression was observed in lncHOXA10 silenced cells (Fig. 4a, b). On the contrary, lncHOXA10 overexpression increased the expression of HOXA10 (Fig. 4c). In conclusion, lncHOXA10 drove the expression of HOXA10. We then detected the role of HOXA10 in liver TIC self-renewal. HOXA10 deficient cells were generated through CRISPR/Cas9 approach and showed impaired sphere formation capacity, indicating the essential role of HOXA10 in liver TIC self-renewal (Fig. 4d).

We then explored whether lncHOXA10 exerted its role through HOXA10. We silenced lncHOXA10 in *HOXA10* knockout cells and examined sphere formation capacity. Upon HOXA10 deletion, lncHOXA10 knockdown had no effect on liver TIC self-renewal, indicating the essential role of HOXA10 in lncHOXA10 function (Fig. 4e). Similarly, lncHOXA10 overexpression also had impaired role in liver TIC self-renewal upon HOXA10 deficiency (Fig. 4f). *HOXA10* knockout cells showed decreased invasion capacity and lncHOXA10 had impaired role upon *HOXA10* knockout, confirming the critical role of HOXA10 in lncHOXA10 function (Fig. 4g, h). Altogether, lncHOXA10 promoted liver TIC self-renewal through HOXA10-dependent manner.

### LncHOXA10 recruited SNF2L to *HOXA10* promoter

**Fig. 5:**
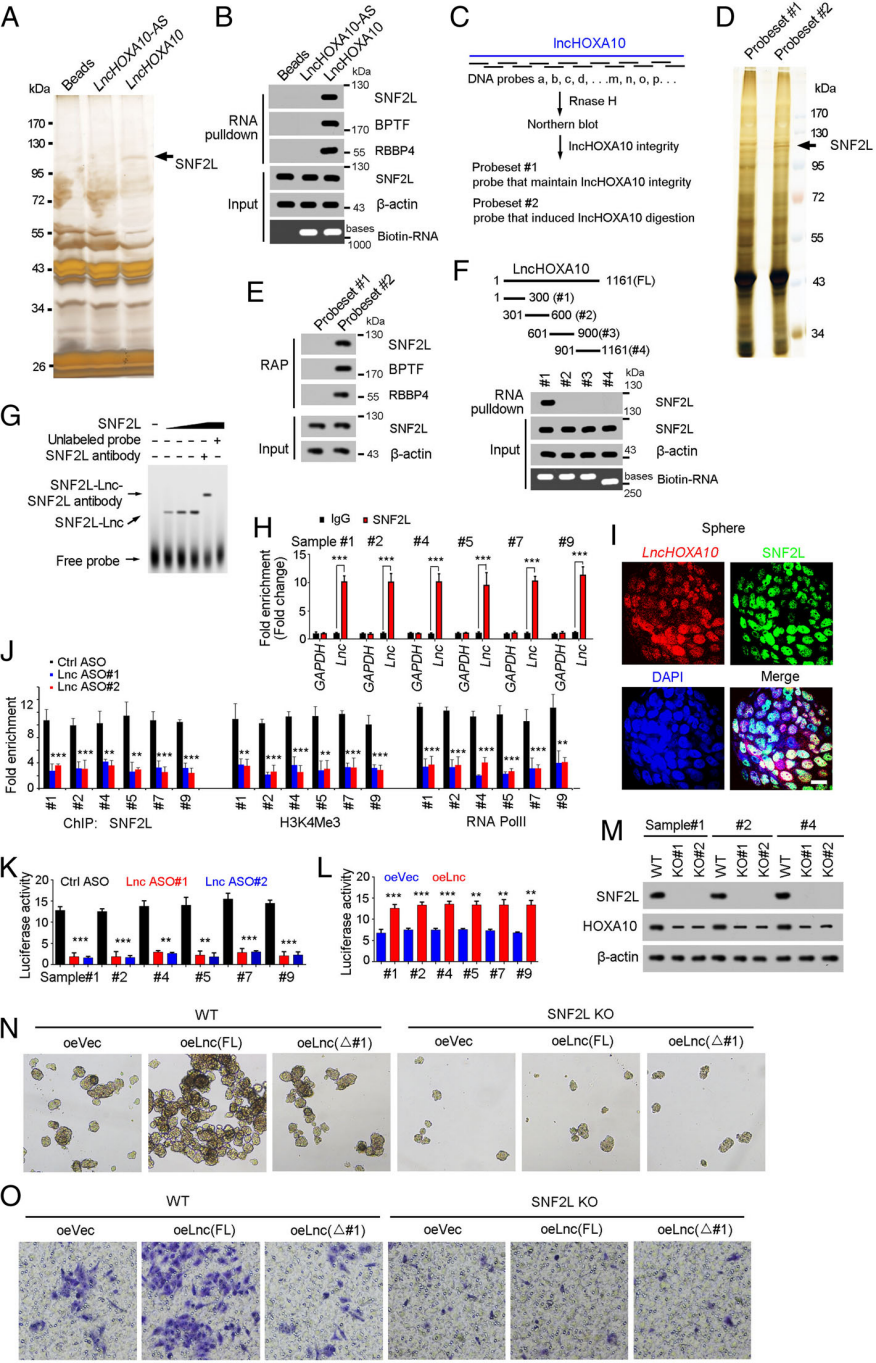
LncHOXA10 interacted with SNF2L. **a** RNA pulldown was performed and the specific band in lncHOXA10 sample was identified as SNF2L by mass spectrum. **b** The combination between lncHOXA10 and SNF2L was examined by RNA-pulldown and Western blot. **c** Diagram showing the procedure of RAP (RNA antisense purification). DNA probes targeting endogenous lncHOXA10 were grouped into Probeset #2. **d** RAP assay was performed and a specific band in Probeset #2 sample was identified as SNF2L. **e** Eluate samples of RAP assay were utilized for SDS-PAGE, followed by Western blot for SNF2L detection. **f** LncHOXA10 truncates were generated (upper panels) and incubated with sphere lysates. The interaction between lncHOXA10 truncates and SNF2L was examined by Western blot (lower panels). **g** RNA electrophoretic mobility shift assay (RNA EMSA) was performed to validate the combination of lncHOXA10 and SNF2L. **h** RNA immunoprecipitation (RIP) assay was performed using oncospheres and enrichment of *lncHOXA10* and *GAPDH* mRNA were examined through realtime PCR. IgG was an antibody control. **i** Double FISH assay showed the co-localization of lncHOXA10 and SNF2L in oncospheres. Scale bars, 10 μm. **j** LncHOXA10 depleted cells were used for SNF2L, H3K4me3 and RNA polymerase II (RNA PolII) ChIP assays, and *HOXA10* promoter enrichment was detected with realtime PCR. (K, L) *HOXA10* promoter was cloned into PGL3 luciferase reporter plasmid, and luciferase activity was measured in lncHOXA10 silenced (**k**) and overexpressing cells (**l**). **m** *SNF2L* knockout cells were generated through CRISPR/Cas9 approach, and the knockout efficiency was confirmed by Western blot. The expression of HOXA10 was also detected by Western blot. **n**, **o** Full length (FL) lncHOXA10 or truncated (△#1) lncHOXA10 was overexpressed in WT and SNF2L KO cells, followed by sphere formation assay (N) and transwell invasion assay (**o**). Data are representative of three independent experiments

We then investigated the molecular mechanism of lncHOXA10 in liver TIC self-renewal and HOXA10 expression. RNA pulldown showed a specific band in lncHOXA10 eluate, which was identified as SNF2L by mass spectrum, indicating the combination of lncHOXA10 and SNF2L (Fig. 5a). The interaction of lncHOXA10 and SNF2L was confirmed by Western blot (Fig. 5b). To further confirm the combination of lncHOXA10 and SNF2L, we performed RAP (RNA antisense purification) assay (Fig. 5c). RAP assay also validated the interaction of lncHOXA10 and SNF2L, which was confirmed by Western blot (Fig. 5d, e). LncHOXA10 truncates were constructed for interaction examination between these truncates and SNF2L. The first region of lncHOXA10 (#1, 1~300 bases) was identified to combine with SNF2L (Fig. 5f), which was confirmed by electrophoretic mobility shift assay (Fig. 5g). RNA immunoprecipitation (RIP) assay also confirmed the interaction of SNF2L and lncHOXA10 (Fig. 5h). The co-localization of lncHOXA10 and SNF2L was also observed in oncospheres through FISH (Fig. 5i). Altogether, lncHOXA10 combined with SNF2L in liver TICs.

SNF2L is a component of NURF chromatin remodeling complex and plays an essential role in transcriptional initiation [37]. Thus we explored the role of lncHOXA10 in HOXA10 transcription initiation. SNF2L combined with *HOXA10* promoter in liver TICs and impaired binding capacity was observed upon lncHOXA10 depletion (Fig. 5j). LncHOXA10 depletion inhibited the activation of *HOXA10* promoter and subsequent transcriptional initiation (Fig. 5j). Luciferase assay also confirmed the critical role of lncHOXA10 in *HOXA10* promoter activation (Fig. 5k, l).

To further explore the role of SNF2L and lncHOXA10 in lncHOXA10 function, we generated *SNF2L* knockout cells through CRISPR/Cas9 approach (Fig. 5m). Similar with lncHOXA10, SNF2L was also required for HOXA10 expression (Fig. 5m). We found SNF2L was required for liver TIC self-renewal and invasion (Fig. 5n, o). Moreover, lncHOXA10 overexpression played an impaired role upon SNF2L knockout (Fig. 5n, o). We also overexpressed truncated lncHOXA10 that couldn’t interact with SNF2L, and found the essential role of SNF2L-lncHOXA10 in lncHOXA10 function (Fig. 5n, o). Altogether, lncHOXA10 recruited SNF2L to *HOXA10* promoter to drive its expression.

### *LncHOXA10*-HOXA10 could be used for liver TIC targeting

**Fig. 6:**
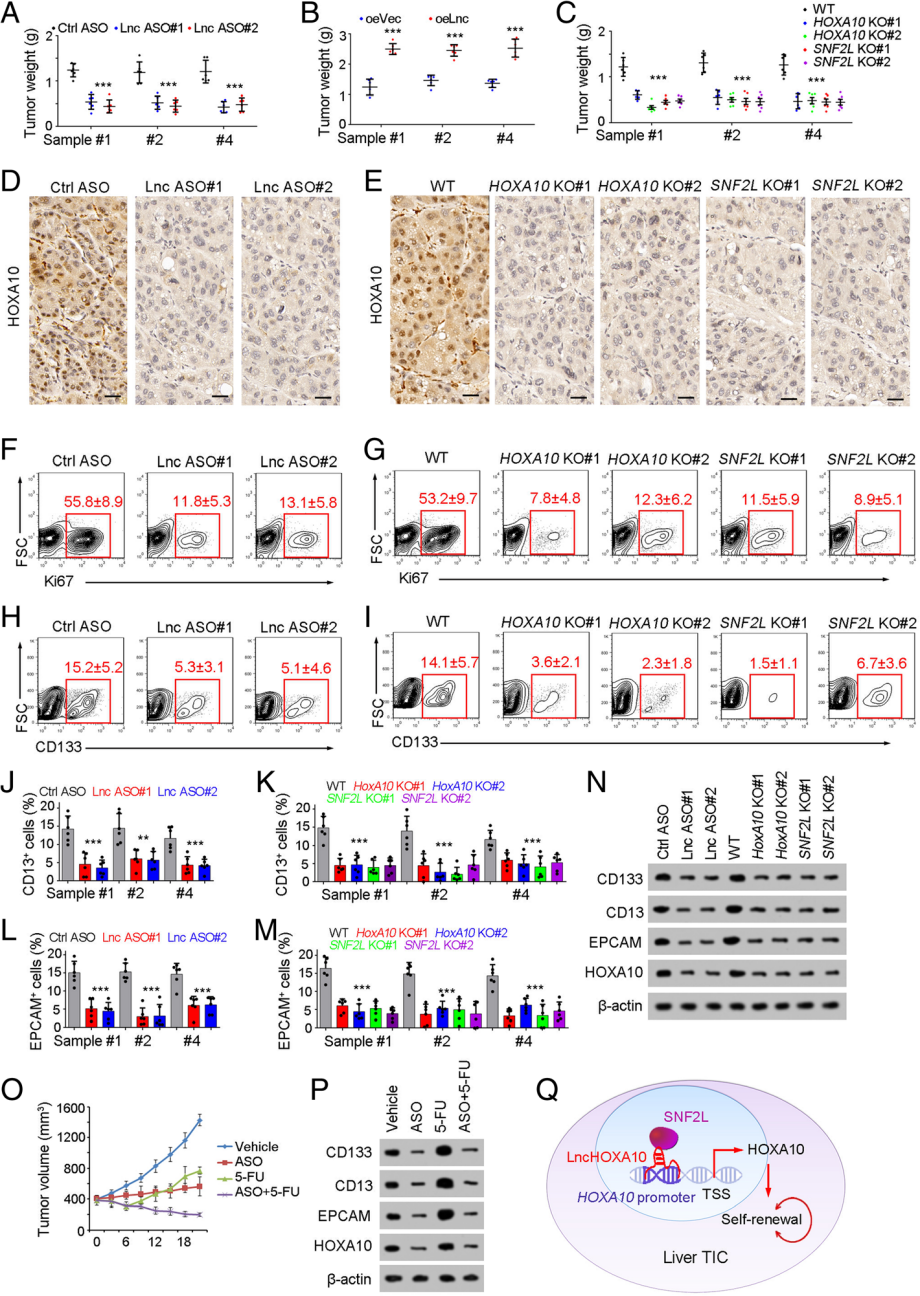
LncHOXA10-SNF2L-HOXA10 pathway could be used for liver TIC targeting. **a**, **b** 1 × 10^6^ lncHOXA10 silenced (ASO, A) or overexpressed (oeLnc, B) cells were injected into BALB/c nude mice. One month later, tumors were obtained and tumor weights were shown as scatter diagram. 6 mice were used for each sample. **c** 1 × 10^6^ indicated cells were used for tumor propagation. Tumor weights were examined 1 month after injection. **d**, **e** The indicated tumors were obtained and immunohistochemistry analyses for HOXA10 expression. **f**, **g** Cell proliferation in the indicated tumors was analyzed by Ki67 staining and FACS. Typical contour diagrams and Ki67^+^ ratios (6 tumors per sample) were shown. **h**, **i** CD133^+^ liver TICs were gated and the proportions of CD133^+^ TICs in indicated tumors were shown. **j**-**m** CD13^+^ and EPCAM^+^ liver TICs were examined in indicated tumors and the ratios of liver TICs were shown. Six tumors were examined for each sample. **n** The indicated tumors were collected and crushed with RIPA lysis buffer, and cell lysate was used for SDS-PAGE, followed by Western blot to detect the expression levels of liver TIC markers. **o**, **p** 400 mm^3^ tumors were treated with lncHOXA10 ASO (ASO) and 5-FU, and tumor volume was measured every 3 days (**o**). Three weeks later, tumors were collected and TIC markers were examined by Western blot (**p**). **q** LncHOXA10 was highly expressed in liver TICs, and recruited SNF2L to *HOXA10* promoter to initiate the expression of HOXA10, finally drove the self-renewal of liver TICs. *P < 0.05, **P < 0.01, ***P < 0.001 by one-tailed Student’s t test. Data are representative of four independent experiments

Finally, we explored the role of lncHOXA10-HOXA10 in liver TIC targeting. Firstly, we examined tumor propagation of lncHOXA10 depleted cells, and found lncHOXA10 played an essential role in liver tumor propagation (Fig. 6a). On the contrary, lncHOXA10 overexpression drove liver tumor propagation (Fig. 6b). Similar with lncHOXA10 silenced cells, *HOXA10* knockout and *SNF2L* knockout cells also showed impaired propagation (Fig. 6c). We obtained the tumors, performed immunohistochemistry assay, and found impaired expression of HOXA10 in lncHOXA10 depleted and *SNF2L* knockout tumors, further confirming the essential role of lncHOXA10/SNF2L in HOXA10 expression (Fig. 6d, e). Ki67 FACS confirmed the essential role of lncHOXA10-SNF2L-HOXA10 axis in liver cancer proliferation (Fig. 6f, g). Decreased populations of CD133^+^, CD13^+^ and EPCAM^+^ TICs were examined in lncHOXA10- SNF2L-HOXA10 inhibited cells (Fig. 6h-m). Western blot also validated the decreased expression of liver TIC markers upon lncHOXA10-SNF2L-HOXA10 inhibition (Fig. 6n). Finally, established tumors were treated with lncHOXA10 ASO, and sustained inhibition was observed, along with decreased expression of TIC markers (Fig. 6o, p). Taken together, lncHOXA10-SNF2L-HOXA10 axis could be used for liver TIC targeting.

In conclusion, we found HOXA10 was highly expressed in liver TICs and identified lncHOXA10 as a modulator for HOXA10 expression. LncHOXA10 interacted with SNF2L and recruited SNF2L to *HOXA10* promoter, drove HOXA10 expression and finally initiated TIC self-renewal.

## Discussion

The self-renewal of liver TICs is precisely regulated, and many signaling pathways are involved in liver TIC regulation. It’s well known that Wnt/β-catenin, Notch and Hedgehog signaling pathways participate in liver TIC self-renewal. Some transcription factors also participate in the regulation of liver TICs, including ZIC2, NOTCH2 and OCT4. Here we identified HOXA10, a member of HOX transcription factor family, drives the self-renewal of liver TICs. Interestingly, the expression of HOXA10 is also regulated by lncHOXA10. LncHOXA10 locates at *HOXA10* promoter and recruits SNF2L to *HOXA10* promoter, which finally activates the transcription initiation of HOXA10.

LncRNAs emerge as critical modulators in many physiological and pathological processes [38]. Here we identified a lncRNA involved in the self-renewal of liver TICs. Of not, lncHOXA10 locates at *HOXA10* promoter. LncRNAs are often co-expressed with their nearby genes, and modulate the transcription initiation of their nearby genes [39]. Here we found lncHOXA10 is co-expressed with HOXA10 in liver cancer and liver TICs. LncHOXA10 regulates the expression initiation of HOXA10 through transcriptional activation by recruiting SNF2L chromatin remodeling complex, adding a new layer for HOXA10 transcriptional regulation. Most recently, some lncRNAs were found to influence the transcription of nearby genes by transcription process itself of lncRNAs [40]. The mutual interaction between HOXA10 and lncHOXA10 transcription processes needs to be further investigated.

As we know, a lncRNA probably targets more than one gene. However, lncHOXA10 has no effluence on liver TIC self-renewal upon *HOXA10* knockout, indicating lncHOXA10 drives liver TIC self-renewal through HOXA10-dependent manner. Several reasons can explain this result. 1. lncHOXA10 locates in specific subcellular position and near from *HOXA10* promoter, and this location limits the number of their target genes. 2. lncHOXA10 targets some other genes, but these genes don’t participate in the self-renewal of liver TICs; 3. some lncRNAs participate in transcriptional regulation through lncRNA transcription process but not lncRNA themselves [40]. The transcriptional process of lncHOXA10 may activate the transcription of their nearby gene HOXA10.

HOX transcription factors play critical roles in many processes, and here we identified HOXA10 as a predominant HOX TF in liver tumorigenesis. HOXA10 exerts an oncogenic role in several tumors, including ovarian clear cell adenocarcinoma, oral squamous cell carcinoma, prostate carcinoma and endometrial adenocarcinoma [23–25, 27]. HOXA10 serves as a good marker for prognosis of various tumors [23, 24, 26], and its high expression leads to enhanced tumor propagation [25, 27]. Overexpression of HOXA10 in hematopoietic cells blocks myeloid/lymphoid differentiation and drives acute myeloid leukemia, indicating a critical role of HOXA10 in differentiation inhibition and tumorigenesis [41]. In human cord blood cells, HOXA10 overexpression also activates the expression of stem-cell specific genes [42]. Despite of the oncogenic role in various tumors and stemness-driving role, the role of HOXA10 in liver tumorigenesis and liver TIC self-renewal is unclear. Here, we detected the critical role of HOXA10 in liver TICs through *HOXA10* knockout and TIC functional assays.

To some extent, tumorigenesis is a process of reprogramming. Many components of chromatin remodeling complexes are dys-regulated along with tumorigenesis, indicating the involvement of chromatin modeling in tumorigenesis. EZH2, a core component of PRC2 modeling complex, is highly expressed in liver cancer and liver TICs [11]. BRG1 and BRM shows BRG1/BRM switch in liver tumorigenesis and liver TICs [10]. Here we found SNF2L, a component of NURF complex, is highly expressed in liver TICs and required for the self-renewal of liver TICs. SNF2L is recruited by lncHOXA10 to *HOXA10* promoter to drive HOXA10 expression. Our finding confirms the importance of chromatin remodeling complex in liver tumorigenesis and liver TICs, and reveals a novel mechanism of HOXA10 expression.

Being in quiescent state and highly expressing drug-resistant pumps like ABCG2, liver TICs are resistant to multiple drugs, and relapse when the drugs are stopped [43]. Here we found 5-FU treatment induced a significant decrease in tumor size at first, but restored and propagated soon (Fig. 6o). In accordance with tumor size, increased expression of TIC markers is observed upon 5-FU treatment, which probably accounts for the ineffective role of long-term 5-FU treatment. Combination of 5-FU and TIC-targeting ASO leads to sustained decrease in tumor size and liver TICs. These observation indicates the importance to combine traditional therapy and TIC-targeting therapy together. In conclusion, lncHOXA10 drives the self-renewal of liver TICs through HOXA10 expression. LncHOXA10-HOXA10 signaling pathway can be targeted for liver TIC elimination.

## Additional files

Additional file 1: **Figure S1.** Un-cropped blot data. (TIF 9968 kb)

Additional file 2: **Table S1.** Raw data of all experimental data. (XLSX 518 kb)

## Abbreviations

ChIP: Chromosome immunoprecipitation
FISH: Fluorescence in situ hybridization
HOXA10: Homeobox A10
ISH: In situ hybridization
LncRNA: Long noncoding RNA
NURF: Nuclear remodeling factor
RAP: RNA antisense purification
RIP: RNA immunoprecipitation
TIC: Tumor initiating cells
